# Changes in quality of life among people with psychosis and their caregivers participating in a community-based rehabilitation intervention in Malawi: A mixed-methods analysis

**DOI:** 10.1371/journal.pmen.0000696

**Published:** 2026-08-25

**Authors:** Mia Buono, Abigail M. Morrison, Hillary Mortensen, Katherine Waddell, Thuy Dao, Bonginkosi Chiliza, Melissa A. Stockton, Bradley N. Gaynes, Kazione Kulisewa, Brian W. Pence

**Affiliations:** 1 Department of Epidemiology, Gillings School of Global Public Health, University of North Carolina at Chapel Hill, Chapel Hill, North Carolina, United States of America; 2 Department of Health Behavior, Gillings School of Global Public Health, University of North Carolina at Chapel Hill, Chapel Hill, North Carolina, United States of America; 3 University of North Carolina Project-Malawi, Lilongwe, Malawi; 4 Department of Psychiatry, Nelson R Mandela School of Clinical Medicine, University of KwaZulu-Natal, Durban, South Africa; 5 Psychiatry Department, Perelman School of Medicine, University of Pennsylvania, Philadelphia, Pennsylvania, United States of America; 6 Department of Psychiatry, University of North Carolina at Chapel Hill School of Medicine, Chapel Hill, North Carolina, United States of America; 7 Department of Psychiatry and Mental Health, Kamuzu University of Health Sciences, Blantyre, Malawi; PLOS: Public Library of Science, UNITED KINGDOM OF GREAT BRITAIN AND NORTHERN IRELAND

## Abstract

Psychotic disorders are a leading cause of disability in Malawi. Community-based rehabilitation (CBR) interventions may improve quality of life (QOL) for people with lived experience of psychosis (PWLE) and their caregivers by addressing functional challenges and promoting empowerment and social reintegration. However, understanding how QOL may evolve over the course of participation in CBR in low-resource settings remains unknown. This analysis examined changes in QOL among PWLE and their caregivers participating in a CBR intervention in Malawi using a mixed-methods approach. We analyzed qualitative and quantitative data from the intervention arm of a two-arm pilot randomized controlled trial comparing a CBR intervention to enhanced usual care in Blantyre, Malawi (NCT06080477). Eligible participants were over 18, diagnosed with schizophrenia spectrum disorder, and had a caregiver willing to participate. Thirty PWLE and 58 caregivers completed interviewer-administered surveys at 6 and 12 months. In-depth qualitative interviews were conducted with a subsample of 12 PWLE and 14 caregivers between 6 and 9 months. Qualitative data were analyzed thematically. Quantitative data were summarized descriptively to assess changes in QOL, functioning, and functional outcomes over time. Qualitative findings highlighted improvements in QOL across multiple domains. PWLE described improvements in mental and physical health, increased independence, and social reintegration that contributed to better QOL. Caregivers reported improved mental health, reduced caregiving burden, and reintegrating into society improved QOL. Quantitative findings aligned with reported themes for PWLE. Mean QOL scores improved from 48.5 at baseline to 52.7 at 12 months. Mean psychosis severity scores decreased from 67.7 to 40.2. Functional outcomes such as employment, community engagement, and monthly income generation improved across the study period. CBR interventions are well-positioned to support psychosis management and improve overall QOL among PWLE and their family members in Malawi, supporting their broader implementation.

## Introduction

Psychotic disorders are among the leading causes of disability globally [1]. When left unmanaged, psychosis can lead to worse functional and mental health outcomes and reduced participation in community and employment activities [2–6]. Worse function and mental health can limit social engagement and daily functioning, subsequently lowering the quality of life for people with lived experience of psychosis (PWLE) and their caregivers [7,8]. While medication management remains an important component of psychosis treatment, antipsychotics alone are unable to comprehensively address the functional, social, and occupational rehabilitative needs of PWLE [9]. Further, there is a growing body of evidence supporting holistic, recovery-oriented interventions [10]. Community-based rehabilitation (CBR) interventions aim to improve long-term quality of life by addressing functional challenges and promoting empowerment, understanding, and social reintegration among PWLE and their caregivers [11–13].

In Malawi, there is a high burden of psychosis and related caregiving needs due to limited health care resources and recovery-oriented treatment options [14,15]. Psychosis treatment in Malawi typically focuses on stabilization and medication administration to primarily reduce positive symptoms of psychosis [16,17]. Therefore, integration of CBR interventions into psychosis care provides a promising avenue for enhancing health outcomes and quality of life for PWLE and their caregivers. However, little is known about how QOL may evolve over the course of participation in CBR among PWLE and their caregivers in Malawi [18].

This study addresses this gap by examining changes in the overall quality of life over a 12-month period among PWLE and their caregivers participating in a CBR intervention in Malawi. We explore both qualitative and quantitative measures of physical health, mental health, and community engagement to determine how the CBR intervention influenced overall quality of life for PWLE and their caregivers.

## Methods

### Ethics Statement

The University of North Carolina Biomedical Institutional Review Board (#23–1665) and the Malawian National Health Sciences Research Committee approved this study (#23/07/4147). All study participants provided written informed consent. The authors had access to information that could identify individual participants during and after data collection. Additional information regarding the ethical, cultural, and scientific considerations specific to inclusivity in global research is included in the Supporting Information (S1 Checklist).

### Design

This paper presents an exploratory mixed-methods analysis of data from the ENHANCE pilot trial. The ENHANCE trial is a two-arm pilot randomized controlled trial that compared enhanced usual care to an adapted community-based rehabilitation (CBR) intervention among community-dwelling individuals with lived experience of psychosis and their caregivers in Malawi (NCT06080477). This analysis explored quantitative and qualitative measures of quality of life among participants with psychosis who received the CBR intervention and their caregivers.

### Study Participants

Participants were recruited from the Queen Elizabeth Central Hospital (QECH) in Blantyre, Malawi, from December 5^th^, 2023, to July 30^th^, 2024. Eligible participants met the following criteria: 1) attended outpatient services, 2) were 18 years or older, 3) had a clinician-confirmed diagnosis of schizophrenia spectrum disorder, 4) were a Blantyre District resident, 5) were not planning to relocate out of Blantyre District in the next 12 months, 6) had a primary caregiver willing to participate in the study, and 7) had current elevated symptoms of poor functioning as demonstrated by one or more of the following: Positive and Negative Symptoms Scale score ≥58, 12-item WHO Disability Assessment Schedule 2.0 score ≥35, and/or Clinical Global Impression Severity score ≥3 (at least mildly ill).

Up to two caregivers per participant were recruited to participate. Eligible caregivers were: 1) a current caregiver for an eligible and consenting patient participant, 2) 18 years or older, 3) a Blantyre District resident, and 4) not planning to relocate out of Blantyre District in the next 12 months.

Eligible and willing individuals provided IRB-approved written informed consent. Sixty people with psychosis and 118 caregivers enrolled in the ENHANCE pilot trial. After completing a baseline research interview, participants, along with their caregivers, were randomized 1:1 to receive enhanced usual care or the adapted CBR intervention. All study participants completed interviewer-administered surveys at 6- and 12-month follow-up. This analysis is restricted to the participants and caregivers assigned to receive the adapted CBR intervention (n = 30 PWLE, n = 58 caregivers). As the goal of the study was to explore whether the intervention improved QOL, usual care participants were excluded.

### Intervention Description

Participants randomized to the intervention received the adapted CBR intervention delivered by the QECH clinical team of psychiatric nurses. The intervention included nurse-delivered home visits over 12 months with decreasing intensity as determined by the nursing team. In the initial phase, focused on stabilization, weekly visits occurred for 2–4 months. In the intermediate phase, focused on rehabilitation, visits occurred biweekly for 4–6 months. In the final phase, focused on the sustainability of lessons learned and transition to independence, visits occurred monthly for 3–4 months. The nurses tailored each phase to address the participants’ unique initial needs and responses to the intervention. CBR modules to meet participant needs included sessions on day-to-day functioning, rehabilitation, and medication use. Modules were delivered with the PWLE and/or caregivers as appropriate. Participants randomized to the enhanced usual care arm received no home-based services, but continued to receive standard clinical care, which was enhanced through the provision of symptomatology and disability scales alongside any relevant clinical recommendations to the clinical team.

### Qualitative Data

Semi-structured interviews were conducted by trained interviewers with 12 PWLE and 14 of their associated caregivers in the intervention arm from December 2024 to January 2025. Participants were generally toward the end of Phase 2 or early in Phase 3 of the intervention (6–9 months) at the time of their participation in the interview. Interviews were conducted by trained interviewers in-person in private places at QECH in Blantyre, Malawi. Interview guides addressed a range of topics, including hypothesized domains of quality of life before and during the intervention. Specifically, participants explained their day-to-day life, physical and mental health, engagement in the community, and what a better quality of life means to them. Participants were asked to reflect on what these domains were like before starting the intervention, 6–9 months previously, and how the domains had evolved over the course of participating in the intervention. The interviews lasted approximately 60–90 minutes and were conducted in Chichewa. Interviews were audio-recorded before transcription. Sessions were then simultaneously translated and transcribed into English.

PWLE and their caregivers who were 6–9 months into CBR were purposively sampled to participate in the qualitative interviews with the goal of achieving thematic saturation [19]. An initial codebook was developed that contained deductive codes based on the interview guides and the goals of the study. The codebook was then iteratively revised and updated to reflect inductive codes, including key themes that were identified, as transcripts were reviewed [20–22].

Coding was conducted in Dedoose 10.0.35 [23] by AM, HM, KW, and TD. Team members all coded the same two transcripts before meeting to discuss any discrepancies and make necessary changes to the codebook. The team then coded an additional two transcripts, addressed discrepancies, and made final changes to the codebook. Each remaining transcript was coded by two team members. Coders met weekly to discuss and address any issues that arose. Once the team finished coding, the team wrote memos to describe and further explore the key themes that had been noted in the coding process [21,22].

### Quantitative Data

#### Measures.

Measures included in the quantitative analysis come from the interviewer-administered structured surveys completed by participants and caregivers at baseline, 6 months, and 12 months. Variables considered relevant to overall quality of life (QOL) among PWLE included measures of QOL, overall functioning, mental health outcomes, and community engagement.

We measured QOL by the Short-Form 8 (SF-8). Normed total scores were used, with higher scores indicating better health [24]. Overall functioning was measured by the 12-item World Health Organization Disability Assessment Schedule (WHODAS). The WHODAS uses a 5-point Likert scale (‘None’ = 1 to ‘Extreme or cannot do’ = 5), with scores ranging from 12-60. Total WHODAS scores were used, with higher scores indicating greater disability [25].

The Patient Health Questionnaire-9 item scale (PHQ-9) was used to measure depressive symptoms. This scale uses a 4-point Likert scale (‘0 days’ = 0, ‘1-7 days’ = 1, ‘8-12 days’ = 2,‘13 or 14 days’ = 3) to assess the frequency of the nine DSM V symptoms of a depressive episode in the prior two weeks, with total scores ranging from 0-27 [26]. The Generalized Anxiety Disorder-7 item scale (GAD-7) was used to measure anxiety symptoms. GAD-7 uses a 4-point Likert scale (‘0 days’ = 0, ‘1-7 days’ = 1, ‘8-12 days’ = 2,‘13 or 14 days’ = 3), to assess the frequency of the seven core symptoms of anxiety in the prior two weeks, with total scores ranging from 0-21 [27].

Community engagement was measured using an open-ended survey question to quantify the number of days participants attended church, market, social activities, and sports games in the past 6 months. Community engagement was analyzed continuously. Demographic variables assessed at baseline included age, sex, marital status, education, employment, and income.

### Statistical Analysis

The focus of the statistical analysis was descriptive to complement the qualitative data, rather than inferential. Accordingly, we summarized baseline sociodemographic, employment, mental health, functional, and community engagement measures using means (SD) for continuous variables and frequencies (percentages) for categorical variables. To evaluate QOL over time, we described employment, mental health, functional, and community engagement measures at 6 and 12 months using means (SD).

## Results

### Study enrollment

Between December 5th, 2023, and July 30th, 2024, 60 PWLE and 118 caregivers were enrolled in the parent pilot trial, of whom 30 PWLE and 58 caregivers were randomized to the intervention arm. For those in the intervention arm, their mean (standard deviation) age was 37.5 (11) years. The majority were male (80.0%), completed secondary school (43.3%), and never married (66.7%). Around half were employed or actively contributing (40%). People were considered actively contributing if they were engaged in some form of activity, including farming, self-employment, homemaking, or a paid job (Table 1).

**Table 1:** Demographics.

	Total(N = 30)
Age (years), mean (SD)	37.5 (11.0)
Gender	
Female	6 (20.0%)
Male	24 (80.0%)
Employment	
Employed/Actively contributing	12 (40.0%)
Unemployed able to work	5 (16.7%)
Unemployed unable to work	3 (10.0%)
Other/Refused	10 (33.3%)
Marriage	
Never Married	20 (66.7%)
Currently Married	5 (16.7%)
Previously Married	5 (16.7%)
Education	
No Schooling	2 (6.7%)
Standard 5	2 (6.7%)
Standard 8	6 (20.0%)
Secondary School	13 (43.3%)
Postsecondary	7 (23.3%)
Total sample: N = 30	

Values are presented as mean (SD) for continuous variables and n (%) for categorical variables. SD = standard deviation; N = number of participants. The study sample includes PWLE who were randomized (N = 30) to receive the community-based rehabilitation intervention in Malawi. Participants were recruited between December 5, 2023, and July 30, 2024.

### Qualitative results

Qualitative results are presented across domains that contribute to QOL among PWLE and caregivers. We first report PWLE and caregiver perspectives on what it would look like to have a better QOL, to highlight how participants conceptualize and imagine improved QOL. Then, we present results across four domains hypothesized from the literature and participant responses that contribute to QOL: mental health, physical health, participation or involvement in community activities, and changes in caregiving. Mental health, physical health, and community engagement results are presented before and during the intervention to capture change over time.

In their qualitative interviews, PWLE and caregivers reported that better QOL included meeting basic needs, connecting with the community, and managing psychosis symptoms. Participants highlighted that over the course of the intervention, domains relevant to better QOL improved. PWLE reported improvements in mental health, physical health, ability to complete daily tasks, and reintegration into society. Similarly, caregivers reported a better QOL during the intervention due to their family members being able to complete daily tasks. Improved independence led to an overall reduced care-taking burden, which allowed caregivers to return to work and reintegrate socially.

#### Perspectives on what it would look like to have a better quality of life.

PWLE and their caregivers were asked what it would look like to have a better QOL for themselves and PWLE specifically. For caregivers, a better QOL focused on remaining healthy, meeting basic needs, and having a stable income-generating job. One caregiver said,

“The first main thing I can talk about is being an individual who is disease-free, as well as an individual who does not frequently fall sick. To me, this would be a better quality of life. In addition to that, I can say that we all know that our day-to-day life requires certain needs in order for our lives to be healthy, as well as to have strength. Individuals need to manage to get food for that day, starting from morning, noon till evening. If a household manages to get these things daily, the people in that household say that they are living a better quality of life.” Caregiver-02

Maintaining overall health and meeting basic needs are central to caregivers’ perceptions of a better QOL. The CBR intervention provides a framework for psychosis management that supports improvements in overall health and meeting basic needs, contributing to enhanced QOL for caregivers.

Caregivers, when describing the overall QOL for their PWLE, described that they want their family members to achieve short-term goals, such as being able to engage in self-care, complete daily chores, and re-engage in work. One caregiver described the importance of short-term goals.

“Doing all the basic things a normal person does when he wakes up in the morning. Things like bathing or doing household chores. Being able to know that at this time I should do this and that. Of course, he does that.” Caregiver-03

Additionally, caregivers noted that a better QOL would encompass their PWLE achieving long-term goals such as building a family and reintegrating fully into society, highlighting the importance of short and long-term goals on QOL.

Similarly to their caregivers, PWLE described meeting basic needs, having a stable job, and the ability to help their family with chores and household income as the key factors for a better QOL.

“To have a stable job, not piecework, but a stable job, and to be able to help my family financially. In that way, I would say I have a better quality of life.” PW-10

PWLE also describe increased community connection with people who treat them respectfully as an important facet of a better life.

“I feel happy when I forget the issues with my friends about visiting friends. Sometimes people treat me like a normal person, they don’t associate me with my illness. Socialize like having friends who take you with that condition and treat you like any other normal person. And another thing is having a stable source of income.” PW-07

Overall, these quotes demonstrate the importance of meeting basic needs and community engagement on the QOL among PWLE. The CBR intervention is uniquely positioned to facilitate PWLE achieving their QOL goals, allowing PWLE to meet their own basic needs and shift focus to longer-term goals and social reintegration.

Finally, caregivers and PWLE described that managed psychosis would be a key driver in a better QOL. Among caregivers, managed psychosis would help them feel freer and more peaceful due to a reduced caregiving burden. Additionally, caregivers stated that managed psychosis would allow PWLE to work, contribute to the household, and engage in self-care, reducing the overall burden of caregiving and allowing caregivers to engage in more community activities. One caregiver described what it would look like for their family member’s psychosis to be well-managed:

“I would be happy knowing that everything at home is okay. I would move freely knowing that both of us are now normal people. Honestly speaking, mental illness is a very serious illness. It is simple when it is someone else’s son who has it, and you get to watch from a distance. But when you have that burden at your home, it is very challenging.” Caregiver-06

Another caregiver described their perspective as:

“I would be at peace since he is cured and he will be able to work again, which means he will be able to take care of our family. I won’t be stressed about what I’m going to do or how things will work out for us. I will be at peace and so will be my family.” Caregiver-11

Among PWLEs, managed psychosis would also contribute to their goals of being able to work, be independent, and contribute financially to their family. One PWLE described:

“First, I will be independent and move out of my parents’ home. And I would also help my family financially, it’s what I really want the most, but because I don’t earn much, it becomes difficult for me to help them out.” PW-10

Taken together, these quotes show that managing psychosis symptoms is integral to achieving better long-term QOL for both PWLE and their caregivers. But a holistic approach to recovery, such as the CBR intervention, is needed to meet the goals and perception of a better QOL among PWLE and caregivers.

#### Mental health.

*Pre-Intervention:* PWLE and caregivers described common mental health concerns as being present before the intervention, including depression, anxiety, worry, and inability to concentrate on work or daily tasks. One caregiver described,

“If we see him alone you can tell that he is stressed or depressed and from there he would leave the house and when you try to talk to him you can see that he is angry” Caregiver-09

Additionally, PWLE noted that their mental health impacted their ability to bathe and complete daily chores. One PWLE highlighted this by describing the challenges of taking care of themselves when they are feeling stressed or depressed.

“PWLE: When I’m stressed or depressed, I can’t concentrate on anything, it doesn’t work.

Interviewer: What about taking care of yourself?

PWLE: I do take care of myself but poorly.” PW-04

Before the CBR intervention, PWLEs’ mental health led to an inability to properly take care of themselves, which subsequently had negative impacts on their mental health and QOL. These results suggest a dynamic, cyclical, and/or compounding relationship between poor mental health and the ability to care for oneself.

Caregivers also described that PWLEs’ condition caused so much internal worry that caregivers often found themselves unable to concentrate at work or forced to leave other obligations to tend to their family members.

“So, I was able to participate but not fully because of the challenging experiences that were occurring most of the time. So, I could not leave my brother in the state he was in. You would hear someone informing you that he has met your brother heading in a certain direction while naked. So, I would drop everything I was doing in order to follow my brother wherever he was going until I found him, even though on other occasions, I could not find him.” Caregiver-02

Another caregiver described how caregiving influenced their mental health,

“We were indeed experiencing stress most of the time. I used to feel a lot of anger.” Caregiver-04

These caregivers describe the mental toll of caregiving and how the demands of caregiving can cause the caregiver’s own mental health and work to suffer. This toll impacts the ability to engage in their own activities, worsening their QOL.

Finally, a few caregivers and PWLE described that they were not experiencing any mental health challenges, such as depression, anxiety, worry, anger, etc., before the intervention, suggesting that mental health challenges, while common, did not impact everyone.

*During the Intervention:* PWLE and caregivers described stark improvements in PWLEs’ mental health over the course of the intervention, leading to an improved QOL. PWLE had improvements in depression, anxiety, anger, and the ability to concentrate. Among the PWLE and caregivers who highlighted PWLE mental health improvements, these improvements were tied to an increased ability to bathe, engage in household chores, complete piecework (picking up various day jobs), and integrate socially. One PWLE described the CBR nurse’s key role in improving their mental health.

“I used to feel stressed, however, when the nurse came, she gave me encouraging [advice] that I should be associating with others by joining a number of groups in the community. In doing so, I will be able to reduce or end the stress I had been experiencing.... Now, it seems as though I have a future, since now I know where to go when I am encountering challenges with the mental illness I experience. Now I know the place to go to get help.” PWLE 01

One caregiver highlighted their family member’s progress.

“I can say that now he is no longer an angry individual because he manages to go out to socialize with other people as well as watch games, like football. We are now able to sit and chat, talking about different things. This shows that he is no longer angry or a worried person.” Caregiver-07

Together, these quotes show how the CBR intervention and nurse support were critical in improving mental health. As PWLE experienced a reduced burden of psychosis symptoms, they were able to reconnect with the community and imagine a future in which they had resources to handle mental health challenges. Improvements in mental well-being, increased community engagement, and a renewed sense of hope collectively contributed to better QOL for PWLE.

Notably, one PWLE said that their anxiety and depression worsened throughout the program. This PWLE did not report improvements in their ability to complete daily tasks or improvements in the overall QOL. This unique example further underscores the connection between mental health symptom improvements (or lack thereof), the ability to complete daily tasks, and subsequent reports of QOL.

Almost all caregivers highlighted that their mental health improved over the course of the intervention. Specifically, caregivers described fewer feelings of worry, depression, anxiety, and anger. These improvements allowed caregivers to resume activities outside of the household without feeling worried about their family members, thereby enhancing their QOL. One caregiver said,

“It has changed a lot because I don’t get angry anymore. When the nurse calls me to say she is coming, no matter how busy I was, I would stop doing whatever I was doing so that we could meet with her. I canceled all my plans to go and meet with her because that’s where all my problems end.” Caregiver-02

Consistent with the perspectives of their PWLE, caregivers emphasized the importance of the CBR intervention in facilitating improvements in PWLEs’ mental health. As PWLEs’ mental health symptoms improved and they were able to re-engage in activities, caregivers experienced a reduced caregiving burden. This alleviation of the mental toll of caregiving allowed caregivers to re-engage in their own activities, improving their overall mental health and QOL.

#### Physical health.

*Pre-Intervention:* Many PWLE and their caregivers described physical health challenges present before the intervention. Among PWLE, the most common physical health challenges included disrupted eating habits, sleep patterns, and headaches. One caregiver describes,

“He used to get sick often, and because of his condition, he wasn’t eating properly or having proper meals.” Caregiver-05

Before the intervention, PWLE suffered physical health challenges due to their psychosis symptoms, which impacted their ability to maintain healthy habits.

Similarly, PWLE noted that their physical health challenges due to psychosis symptoms prevented them from completing daily household chores.

“I was not managing to work, as well as that I was not able to participate in taking care of our household.” PWLE-11

Many PWLE faced challenges engaging in daily tasks because of their physical health.

In addition to physical health challenges among PWLE, one caregiver described how their own physical health had been impacted by their family member’s condition.

“Since we used to think a lot about how he was living his life or the things he was doing, we used to feel a lot of pressure, emotionally. We were constantly worrying as well as lacking inner peace. The outcome was that we ended up losing a lot of weight, since our physical health and well-being were being negatively impacted.” Caregiver -04

In this way, the burden of caregiving took a toll on physical health. Due to the consistent worry and demands of caregiving, caregivers’ physical health and overall QOL suffered.

*During the Intervention:* PWLE and caregivers both noted some physical health improvements over the course of the CBR intervention. Among PWLE, eating habits, sleep patterns, and the ability to complete daily chores improved. One caregiver said of their family member,

“In regard to his physical health wellbeing, it has indeed changed. Nowadays, he is able to wash his clothes, or he manages to bathe on his own. He manages, now, to chat with his friends appropriately. He also manages to eat freely, now, while in the past, when his mental illness condition was still severe, it was even challenging for him to manage to eat freely. He used to be selective about his food, then.” Caregiver-04

The caregiver then noted improvements to their own physical health,

“We [PWLE’s family members] no longer live in fear. Even our [PWLE’s family members] physical health well-being has improved.” Caregiver-04

These physical health improvements allowed PWLE to complete basic needs and reintegrate socially, subsequently improving their overall QOL. Additionally, progress made by PWLE decreased worry among caregivers, improving caregivers’ physical and mental health, and thus their overall QOL.

Some respondents described not experiencing any improvement, but all participants (both PWLE and caregivers) who mentioned no improvement described having no physical health challenges before the intervention. One caregiver described,

“It has stayed the same. Sometimes he gains weight, and sometimes he loses weight. And physically, he is just fine, nothing has changed.” Caregiver-09

Therefore, the intervention appeared to support improvements in physical health among PWLE and caregivers, which improved overall QOL. Where improvements did not occur, the CBR intervention helped support the maintenance of physical health.

#### Participation or involvement in community activities.

*Pre-Intervention:* PWLE and their caregivers both reported limited involvement in community activities prior to the intervention. PWLE described community perception as limiting their community involvement. When asked about being involved in community or social activities, one PWLE said,

“No, I have the feeling that everyone knows about my illness and the story behind it.” PW-07

Among caregivers, the time burden of caregiving, emotional distress, and community perception of their family members limited community involvement. One caregiver noted,

“I had been selected to be the chairperson at church. So, I was failing to do my duties as the chairperson. So, it seemed as though I had failed to perform my duties as a chairperson just because I was not having the time to attend to these duties, due to having a son who was unwell at home.” Caregiver-12

Similarly, another caregiver said,

“Yes, because of his behavior, I don’t have good relationships with people… Because of his behavior, he would fight with people, and that was one of the things that made me anxious, and I was very depressed. I couldn’t participate in group activities because when I did, people disrespected me. Because of that, I decided to just stay at home. I would go to church and then back home.” Caregiver-13

Persistent community stigma and the demands of caregiving isolated caregivers and PWLE from participating in community life in ways they may have wanted to, negatively affecting their QOL.

While many caregivers described challenges integrating into community activities, some respondents were happy with their level of community engagement before the intervention.

“Yeah, I mean... particularly in high school, I was involved in charity work. Maybe going to local schools, and educating, helping people in Math. Playing soccer. Doing exercises and other things like that. So, I have been involved in community and charitable work. Right now, friends, seeing friends, and things like that. I have always been there. It’s just a continuation.” Caregiver-08

These quotes show that while some caregivers were disengaged from the community before the intervention, others had maintained strong community involvement. Nevertheless, challenges with community involvement were a notable concern for many PWLE and caregivers.

*During the Intervention*: PWLE and caregivers reported improvements in their ability to re-integrate into the community over the course of the intervention. Specifically, caregivers described that the nurse CBR visits increased their PWLE’s ability to bathe and complete daily chores, reducing the time burden of caregiving. Caregivers noted that these improvements decreased their emotional distress around leaving their PWLE to go to work, the market, or social activities, improving engagement in the community and QOL.

“It has changed because the nurse would talk to him as a patient and tell him what to do, and then she would talk to us as guardians and tell us what to do. She would tell him, ‘You need to do this and that. When you wake up in the morning, try to be active and help with household chores. Don’t oversleep.’ Because of that, it has helped him improve, and now we are all on the same page. That’s why we can move freely to attend or participate in community activities.” Caregiver-05

Another caregiver reported,

“Things have changed because way back, I was unable to move freely, and I couldn’t be gone for long, considering the situation and that I had left a patient at home. But now I can go to church and leave him at home with the children, and he made the home just fine.” Caregiver-11

Overall, these quotes reflect a consistent theme that the intervention facilitated PWLEs’ ability to complete daily tasks and chores independently. This newfound autonomy reduced caregivers’ physical and mental burden, allowing them to re-engage in community activities. In turn, the opportunity to reconnect with their social circles improved the QOL among caregivers.

Additionally, PWLE reported improvements in community engagement, quality of their social interactions, and QOL. One PWLE described the impacts of CBR:

“It [CBR] has changed in a way that they have given me back my position at church, so I have started going to church. I’m a Sunday school teacher.” PW-02

Increased community involvement among PWLE and caregivers not only improved QOL among respondents but also shifted community perception of people living with psychosis. One caregiver said,

“Since I’m no longer depressed, I forgave them, and I chat with them. When they say that “your son has improved,” now I tell them, yes, God’s time is the best. When some people say negative things about him, I don’t pay attention to them.” Caregiver-13

One PWLE described,

“There are now perceiving my mental illness differently, because in the past I was not managing to associate with other people appropriately. … Since I have been taught by the nurses, now, I am able to associate with people more frequently.” PW-11

In combination, these quotes demonstrate that the CBR intervention not only improved engagement in community life for both PWLE and caregivers but also improved community perceptions of those with psychosis. These changes may foster further community engagement for both PWLE and caregivers and improve QOL.

#### Changes in caregiving burden.

Caregivers highlighted that the time and burden of caregiving decreased during the intervention. This reduced caregiving burden improved QOL through improving the caregiver’s ability to work, engage in business endeavors, and integrate into the community.

“Now, most of the time, I am no longer following up or monitoring him, in regard to how he is behaving, since he no longer behaves in a disruptive way... so I see that everything is now progressing well...” Caregiver-12

Another caregiver said,

“Yeah, my ability has changed, yes, I can say that in the past, before joining the CBR program, there were other times when I was not able to go to work because of the condition my brother was in. Sometimes, I would be at work, and they would call me. In so doing, it meant that my work would stop. So, since the time I started participating in the CBR program, I cannot lie that there is a single day on which I was called to resolve issues or failed to go to work because of something my brother had done. Now everything is progressing on well.” Caregiver-02

Additionally, PWLEs’ ability to assist with household chores, support the family business, and re-engage in piecework reduced the overall and financial burden of caregiving, improving QOL for PWLE and caregivers. One caregiver described this process for their family member.

“Things have changed because now he knows that he has to work and provide for his family. He has improved because at first, he couldn’t do any kind of work, but now we work together at the farm, and he is able to do piece work carrying sand. Furthermore, we run a business at home, we sell charcoal, and he used to forget to change, and we had to help him, but now he can give customers change without our help.” Caregiver-11

In supporting PWLE to contribute financially to their families, the CBR intervention had a positive impact not only on PWLE themselves, but also on reducing the financial strain on the caregivers.

### Quantitative results

Responses to the parent trial’s quantitative surveys align with the findings from the qualitative interviews. At baseline, participants in the intervention arm reported high levels of symptoms of psychosis, depression, and anxiety, and low levels of employment (Table 2). Over the course of the intervention, measures of physical health, mental health symptoms, and overall QOL all improved. Community engagement, reflected in days spent going to church, the market, and sports games, increased from baseline to 6 months and then declined, but remained higher than baseline at 12 months. From baseline to 12 months, individual income earned per month increased from around 19,000 (2100) to 44,000 (7400), and the proportion of the PWLE who were employed or actively contributing to the household increased from 40% at baseline to 86% at 12 months (Table 2).

**Table 2:** Functional, mental health, and community engagement variables over time.

	Baseline	6 months	12 months
Mean (SD), % (n/N)	(N = 30)	(N = 29)	(N = 29)
**Overall QOL**			
SF-8 Total Score	48.48 (8.30)	50.62 (10.24)	52.68 (5.65)
**Mental Health**			
PHQ-9 Total Score	4.63 (4.38)	3.28 (3.06)	2.17 (2.38)
GAD-7 Total Score	3.43 (4.11)	2.97 (3.63)	1.31 (1.71)
**Physical Health**			
WHODAS Total Score	20.43 (9.21)	16.17 (5.16)	14.41 (3.62)
PANSS Total Score	67.70 (30.69)	43.34 (12.56)	40.24 (8.81)
Community Engagement*			
Days attended church	14.90 (41.60)	12.97 (10.67)	13.69 (12.66)
Days attended market	59.97 (84.58)	68.48 (76.91)	52.34 (61.44)
Days engaged in social activity	21.40 (52.32)	53.03 (71.82)	44.97 (52.43)
Days attended sports game	1.77 (5.69)	15.83 (47.19)	7.97 (20.74)
Monthly income**	18.75 (21.09)	22.19 (21.0)	44.05 (74.04)
Employed/Actively contributing	40% (12/30)	79% (23/29)	86% (25/29)

*Days attended in the past 6 months.

** Malawian kwacha (x1000).

Values are presented as mean (SD). Sample sizes were N = 30 at baseline, N = 29 at 6 months, and N = 29 at 12 months. One participant in the intervention arm passed away during the study period.

## Discussion

To our knowledge, this is the first paper to explore how a CBR intervention may improve overall QOL among PWLE and caregivers in Malawi. Qualitative and quantitative data suggest that the ENHANCE CBR intervention supported improvements in mental health, physical health, community engagement, and overall QOL among PWLE. PWLE described during qualitative interviews how symptom improvements, nurse support, and hope led to better physical and mental health. These improvements allowed PWLE to participate in more day-to-day tasks and contribute more to their overall community, improving their QOL. Quantitative survey responses aligned with qualitative findings, demonstrating improvements in mental health, physical health, functioning, social engagement, and QOL. Caregivers also reported improvements both among their PWLE and their own mental and physical health, social engagement, and QOL. Together, these findings suggest that the CBR intervention is positioned to promote meaningful improvements in QOL among PWLE and their caregivers in Malawi.

Our results complement other studies that have demonstrated the importance of recovery-oriented approaches to mental health management to promote better health and QOL outcomes [10]. These studies emphasize the role of CBR in addressing functional needs, promoting empowerment, understanding, and social inclusion [11–13,18]. Consistent with the literature, our exploratory mixed-methods findings indicate that the ENHANCE CBR fostered improvements across these domains. However, these improvements cannot be interpreted causally. CBR has also been shown to improve caregivers’ QOL through reduced caregiving burden [28]. These findings align with qualitative reports from family members describing how the intervention reduced stress, improved mental and physical health, and fostered reintegration into their social networks. Additional studies exploring CBR interventions should measure caregiving burden as an important aspect of QOL among caregivers.

We explored how well the quantitative survey measures represent participant reports of functioning, physical health, mental health, and overall QOL. Quantitative measures for functioning, physical health, and mental health aligned well with PWLE reports of psychosis symptoms and the effects of symptoms on daily life. The quantitative measure of QOL, the SF-8, captured the importance of general health on overall QOL. However, the SF-8 does not measure the broader range of long-term needs and goals expressed by PWLE as important to QOL. PWLE emphasized that meeting basic needs, engaging in work, and community reintegration are central to their perceptions of QOL. While our measures of community engagement, demographics, and functioning provided evidence for these domains, future studies should incorporate additional questions or measures to fully capture QOL among PWLE, as the SF-8 does not capture all QOL domains.

Several features of the ENHANCE CBR intervention may explain the notable QOL improvements among PWLE and caregivers. Specifically, the combination of continued nurse support, structured home visits, engagement of family members, frequent needs and goal-setting activities, and tailored recovery modules likely fostered improvements across each unique recovery domain. Additionally, the intervention characteristics may have been particularly well-suited to improve the QOL among this population, given the immense caregiving burden and limited mental health resources for psychosis management.

These findings are important in Malawi. Where mental health resources are available, psychosis treatment typically focuses on medication management of symptoms [16,17]. Relapse while on medication alone, without more holistic support, is still common, due to medication nonadherence and challenges reintegrating into society. A recovery-oriented approach, as demonstrated by our findings, supports the overall well-being of PWLE and their family members. This approach may reduce medication non-adherence and relapse among PWLE by addressing functional, mental, and social needs that support and maintain recovery. Thus, CBR offers a unique opportunity to improve the health and well-being of PWLE and their family members in Malawi and other resource-constrained settings.

All qualitative data were collected after participants had completed 6–9 months of the CBR intervention. As a result, participants were asked to recall QOL domains before the intervention, which may have introduced recall bias. While this is a concern, quantitative findings substantiate qualitative reports. Notably, baseline measures of functioning, mental health, and community engagement align with participants’ pre-intervention accounts; observed improvements in these outcomes over time reflect qualitative reports during the intervention.

Overall, this paper highlights the value of CBR interventions for psychosis management in Malawi and similar low-resource environments. Our findings show that both PWLE and their family members who are currently engaged in outpatient medical management benefit from a complementary recovery-oriented approach that supports improvements across functional, mental, and social domains. These results support the need to expand CBR interventions to improve QOL for individuals living with psychosis and their family members.

## Supporting information

S1 Checklist Inclusivity in Global Research. (DOCX)

## References

[1] Disease GBD, Injury I, Prevalence C. Global, regional, and national incidence, prevalence, and years lived with disability for 328 diseases and injuries for 195 countries, 1990-2016: a systematic analysis for the Global Burden of Disease Study 2016. Lancet. 2017;390(10100):1211–59. 28919117 10.1016/S0140-6736(17)32154-2 PMC5605509

[2] Yu M, Tan Q, Wang Y, Xu Y, Wang T, Liu D, et al. Correlation between duration of untreated psychosis and long-term prognosis in chronic schizophrenia. Front Psychiatry. 2023;14:1112657. doi: 10.3389/fpsyt.2023.1112657 36873212 PMC9978092

[3] Heilbronner U, Samara M, Leucht S, Falkai P, Schulze TG. The longitudinal course of schizophrenia across the lifespan: Clinical, cognitive, and neurobiological aspects. Harv Rev Psychiatry. 2016;24(2):118–28. doi: 10.1097/HRP.0000000000000092 26954596 PMC5079232

[4] Marshall M, Lewis S, Lockwood A, Drake R, Jones P, Croudace T. Association between duration of untreated psychosis and outcome in cohorts of first-episode patients: a systematic review. Arch Gen Psychiatry. 2005;62(9):975–83. doi: 10.1001/archpsyc.62.9.975 16143729

[5] Buckley PF, Miller BJ, Lehrer DS, Castle DJ. Psychiatric comorbidities and schizophrenia. Schizophr Bull. 2009;35(2):383–402. doi: 10.1093/schbul/sbn135 19011234 PMC2659306

[6] Weittenhiller LP, Mikhail ME, Mote J, Campellone TR, Kring AM. What gets in the way of social engagement in schizophrenia? World J Psychiatry. 2021;11(1):13–26. doi: 10.5498/wjp.v11.i1.13 33511043 PMC7805250

[7] Shibre T, Kebede D, Alem A, Negash A, Deyassa N, Fekadu A, et al. Schizophrenia: illness impact on family members in a traditional society--rural Ethiopia. Soc Psychiatry Psychiatr Epidemiol. 2003;38(1):27–34. doi: 10.1007/s00127-003-0594-7 12563556

[8] Killaspy H, White S, Lalvani N, Berg R, Thachil A, Kallumpuram S, et al. The impact of psychosis on social inclusion and associated factors. Int J Soc Psychiatry. 2014;60(2):148–54. doi: 10.1177/0020764012471918 23399990 PMC4107835

[9] Bjornestad J, Lavik KO, Davidson L, Hjeltnes A, Moltu C, Veseth M. Antipsychotic treatment - a systematic literature review and meta-analysis of qualitative studies. J Ment Health. 2020;29(5):513–23. doi: 10.1080/09638237.2019.1581352 30862219

[10] Kane JM, Robinson DG, Schooler NR, Mueser KT, Penn DL, Rosenheck RA, et al. Comprehensive versus usual community care for first-episode psychosis: 2-year outcomes from the NIMH RAISE Early Treatment Program. Am J Psychiatry. 2016;173(4):362–72. doi: 10.1176/appi.ajp.2015.15050632 26481174 PMC4981493

[11] de Wet A, Pretorius C. Perceptions and understanding of mental health recovery for service users, carers, and service providers: A South African perspective. Psychiatr Rehabil J. 2021;44(2):157–65. doi: 10.1037/prj0000460 33151709

[12] Parker JS. Developing the philosophy of recovery in South African mental health services. Afr J Psychiatry (Johannesbg). 2012;15(6):417–9. doi: 10.4314/ajpsy.v15i6.51 23160615

[13] Parker J. Recovery in mental health. S Afr Med J. 2014;104(1):77. 24575463 10.7196/samj.7732

[14] Barnett BS, Kusunzi V, Magola L, Borba CPC, Udedi M, Kulisewa K, et al. Description of the inpatient population and care received at a psychiatric unit in Lilongwe, Malawi. Int J Cult Ment Health. 2018;11(4):574–82. doi: 10.1080/17542863.2018.1448424 32863863 PMC7450978

[15] Kaminga AC, Myaba J, Dai W, Liu A, Chilale HK, Kubwalo PF, et al. Association between referral source and duration of untreated psychosis in pathways to care among first episode psychosis patients in Northern Malawi. Early Interv Psychiatry. 2020;14(5):594–605. doi: 10.1111/eip.12885 31657157 PMC7496144

[16] Kauye F. Management of mental health services in Malawi. Int Psychiatry. 2008;5(2):29–30. doi: 10.1192/s1749367600005531 31507932 PMC6734821

[17] Kauye F, Chiwandira C, Wright J, Common S, Phiri M, Mafuta C, et al. Increasing the capacity of health surveillance assistants in community mental health care in a developing country, Malawi. Malawi Med J. 2011;23(3):85–8. 23448002 PMC3588564

[18] Chatterjee S, Patel V, Chatterjee A, Weiss HA. Evaluation of a community-based rehabilitation model for chronic schizophrenia in rural India. Br J Psychiatry. 2003;182:57–62. doi: 10.1192/bjp.182.1.57 12509319

[19] Saunders B, Sim J, Kingstone T, Baker S, Waterfield J, Bartlam B, et al. Saturation in qualitative research: exploring its conceptualization and operationalization. Qual Quant. 2018;52(4):1893–907. doi: 10.1007/s11135-017-0574-8 29937585 PMC5993836

[20] Gibbs GR. Analyzing Qualitative Data. London: SAGE Publications Ltd. 2018.

[21] Braun V, Clarke V. Thematic analysis. APA handbook of research methods in psychology, Vol 2: Research designs: Quantitative, qualitative, neuropsychological, and biological. Washington, DC, US: American Psychological Association. 2012. p. 57–71. doi: 10.1037/13620-004

[22] Saldaña J. The coding manual for qualitative researchers: 3rd edition. London: 1 Oliver’s Yard, 55 City Road. 2016.

[23] Dedoose (Version 9.2.5). SocioCultural Research Consultants Lwdc [Computer software]. 2024.

[24] Ware JE, Kosinski M, Dewey JE. How to score and interpret single-item health status measures: a manual for users of the SF-8™ Health Survey. Lincoln, RI: Quality Metric Incorporated. 2001.

[25] WHO. Measuring health and disability: Manual for WHO Disability Assessment Schedule (WHODAS 2.0). Geneva: World Health Organization. 2010.

[26] Kroenke K, Spitzer RL. The PHQ-9: A new depression diagnostic and severity measure. Psychiat Ann. 2002;32(9):509–15.

[27] Chibanda D, Verhey R, Gibson LJ, Munetsi E, Machando D, Rusakaniko S, et al. Validation of screening tools for depression and anxiety disorders in a primary care population with high HIV prevalence in Zimbabwe. J Affect Disord. 2016;198:50–5. doi: 10.1016/j.jad.2016.03.006 27011359

[28] Ghebre M, Hepperlen R, Biggs J, Lunda E, Mwandileya W, Rabaey P, et al. Community-Based Interventions Improve the Quality of Life for Children Living With Disabilities, Zambia. Child Care Health Dev. 2026;52(1):e70203. doi: 10.1111/cch.70203 41499214 PMC12778894

